# Incision of the Internal Membrane Under an Endoscope for Advanced Organized Chronic Subdural Hematoma: A Case Report

**DOI:** 10.7759/cureus.83213

**Published:** 2025-04-29

**Authors:** Shoko M Yamada, Yusuke Tomita, Naotaka Iwamoto, Shiori Nishimura

**Affiliations:** 1 Neurosurgery, Teikyo University Mizonokuchi Hospital, Kawasaki, JPN; 2 Neurosurgery, Shizuoka Welfare Hospital, Shizuoka, JPN; 3 Nursing, Teikyo University Mizonokuchi Hospital, Kawasaki, JPN

**Keywords:** burr-hole, chronic subdural hematoma, endoscope, inner membrane, organized

## Abstract

Craniotomy with membranectomy is recommended for advanced organized chronic subdural hematomas (CSDH) that do not resolve with burr-hole irrigation and drainage. Nevertheless, the procedure remains controversial because of its potential complications, which include postoperative acute subdural hematomas and epileptic seizures. At present, for advanced organized CSDH, there is no clear consensus on whether complete membrane removal or incisions are the optimal management. We have obtained good results by performing a small craniotomy and making incisions in the thick, organized inner membrane with a sharp blade, without dissecting the inner membrane. Here, we report a case of organized CSDH that was successfully resolved by the same procedure performed with an endoscope through the burr-hole. An 81-year-old man was referred to our hospital with twice-recurrent CSDH after two burr-hole surgeries, accompanied by persistent left-sided motor weakness. A magnetic resonance imaging revealed an organized inner membrane, so an endoscopic incision of the inner membrane was performed through the previously opened burr-hole. After the clots in the hematoma cavity were cleanly removed, incisions were made randomly in the inner membrane using a sharp blade, with care not to damage the cortex, until pulsation of the brain tissue was observed endoscopically. A follow-up head computed tomography scan on the third postoperative day confirmed brain expansion. The patient recovered completely from the left-sided paralysis and was discharged home on the 20th postoperative day. In an advanced organized CSDH, the brain will not expand without treatment of the inner membrane, and CSDH will invariably recur. Detaching the hard inner membrane is not necessary, and once multiple incisions are made with a scalpel and the brain pulsation is confirmed, expansion can be expected after drainage of the hematoma cavity over multiple days. Several reports of successful treatment for organized CSDH have been reported, but absolute treatment has not yet been established. Incision of the inner membrane in endoscopic burr-hole surgery is a viable treatment option, with the need for large-scale pragmatic studies to prove safety and efficacy.

## Introduction

Advanced organized chronic subdural hematomas (CSDH) are observed in children and the elderly. Burr-hole irrigation and drainage of the hematoma are frequently ineffective because the brain bulge is interrupted by a poorly extensible, thick inner membrane; this leads to CSDH recurrence without brain re-expansion to its original state. An optimal treatment for organized CSDH has not been well established. However, craniotomy is generally the preferred approach when a solid inner membrane is present and requires direct treatment [1,2]. For an advanced organized CSDH, craniotomy with membranectomy has been performed [2]. However, it has been associated with postoperative epileptic seizures, acute subdural hematoma, and subarachnoid hemorrhage [3,4]; death due to acute postoperative hemorrhage has also been reported [5]. In place of membranectomy, we have used incisions of the hard inner membrane after craniotomy for advanced organized CSDH, with positive results. Here, we report on a case in which the same procedure was performed under endoscopy with equivalent results to craniotomy.

## Case presentation

**Figure 1:**
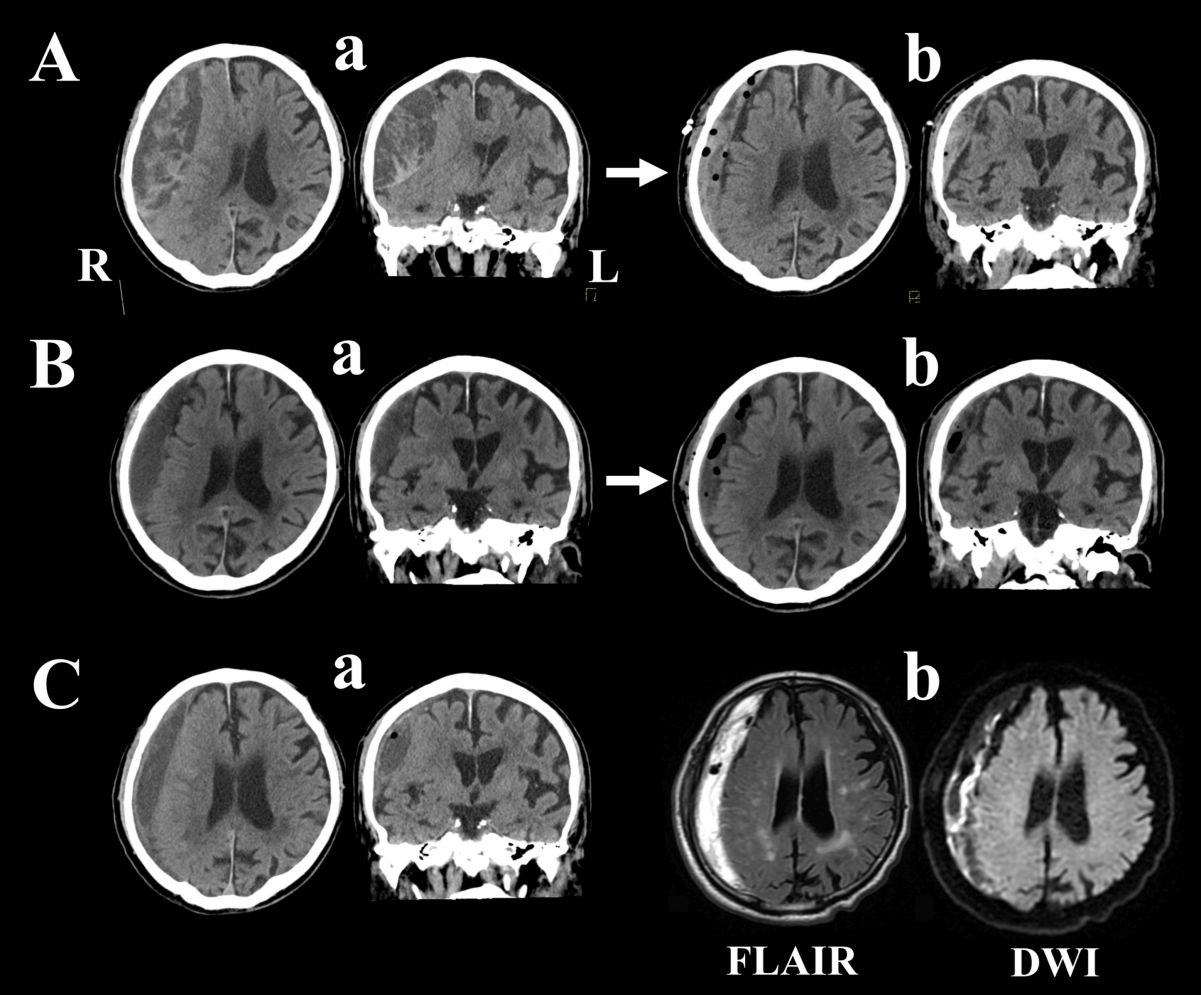
Before endoscopic surgery Aa: A computed tomography (CT) scan before the first surgery showed a thick subdural hematoma with heterogeneous density, causing strong compression to the cerebral cortex and midline shift; b: burr-hole irrigation and drainage contributed to a reduction of the hematoma volume, resulting in the release of brain compression and improvement of the midline shift. Ba: Head CT 20 days after the first surgery revealed re-accumulation of subdural hematoma and compression of the brain with midline shift; b: second irrigation and drainage were performed, contributing to a decrease in the hematoma volume and release of brain compression. Ca: Thirty days after the second surgery, a CT revealed further accumulation of the subdural hematoma, compressing the cerebral cortex; b: magnetic resonance imaging revealed heterogeneous signals along the edge of the cerebral cortex within the hematoma cavity on both fluid-attenuated inversion recovery and diffusion-weighted images, suggesting the presence of an organized inner membrane. FLAIR: fluid-attenuated inversion recovery; DWI: diffusion-weighted MRI

An 81-year-old man was admitted to the hospital with progressive left-side motor weakness and disorientation two months after sustaining a minor head injury. Manual muscle test (MMT) showed a strength of 3-/5 in both the left upper and lower limbs. Head computed tomography (CT) showed a heterogeneous right CSDH, suggesting a septal formation in the hematoma cavity (Figure 1Aa). Burr-hole irrigation and drainage of the hematoma were performed (Figure 1Ab), and left-side muscle strength improved to MMT 4-/5; however, the patient was still unable to walk. Left-side muscle strength gradually weakened over the first week after surgery, to MMT 3+/5 at 20 days after surgery. Head CT revealed recurrence of the CSDH (Figure 1Ba), and burr-hole irrigation and drainage were re-performed using the original burr-hole from the first surgery (Figure 1Bb). Muscle strength recovered to MMT 4+/5, but the patient still had difficulty walking and was transferred to a rehabilitation hospital 10 days after the second operation. Despite rehabilitation, his muscle strength progressively declined in the hospital, and 20 days later, the patient was referred to our hospital with a left MMT of 3/5. Head CT showed further recurrence of CSDH (Figure 1Ca), and magnetic resonance imaging (MRI) revealed organized thrombi and thickened inner membrane in the hematoma cavity (Figure 1Cb). For the third surgery, an endoscopic procedure was performed through the burr-hole opened in the previous surgery.

**Figure 2:**
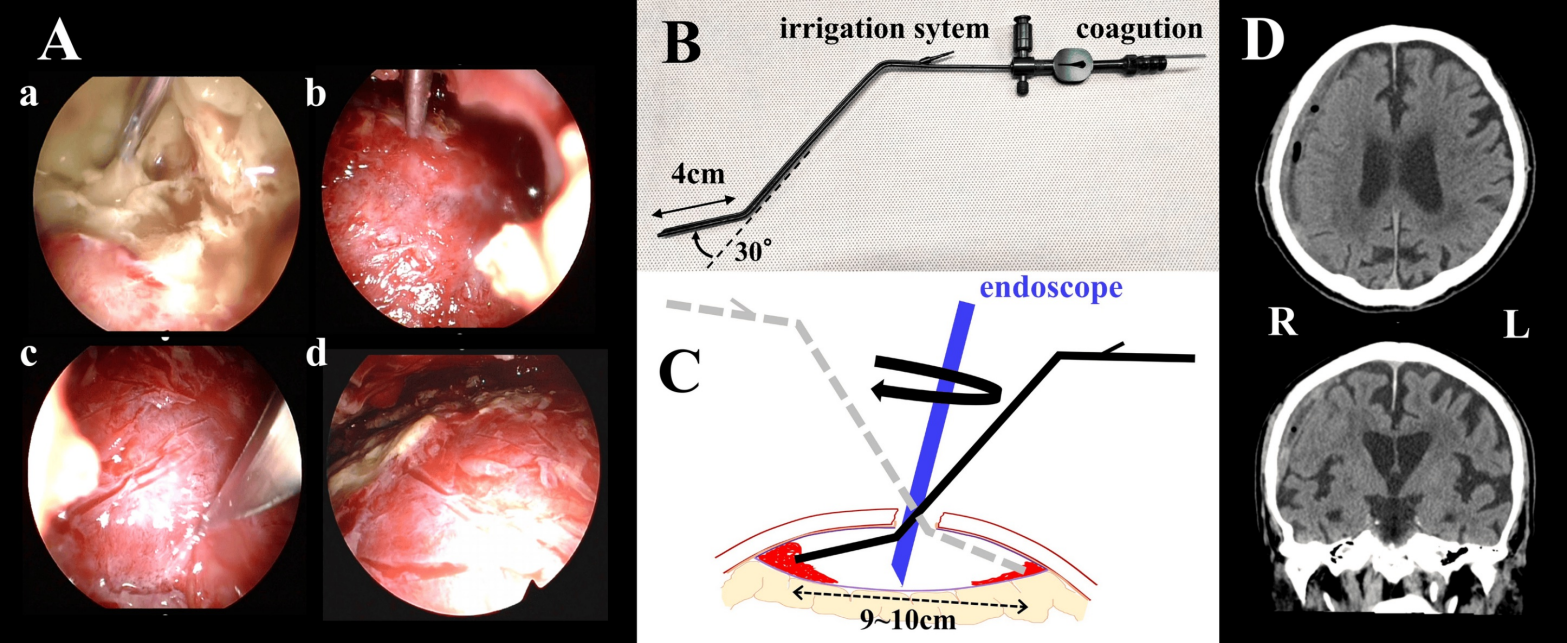
Endoscopic surgery and the result Aa: Jelly-like white clots were aspirated by suction as much as possible; b: the hard inner membrane was exposed, no brain pulsations were recognized due to this hard membrane; c: incisions were made gently with a sharp blade, without damaging the deeper inner membrane or stiffened pia mater on the brain surface; d: when multiple incisions had been made in the inner membrane, simultaneous brain expansion and pulsation were observed. B: The suction device used to remove clots deep in the hematoma cavity was fabricated by the author in an original shape, with a 30°bend at the 4 cm tip; the device possesses irrigation and coagulation capabilities. Figure 2B is created by the author, Shoko M. Yamada. C: The 4 cm tip can be inserted simultaneously with the endoscope into the hematoma cavity without damaging brain tissue and can be rotated 180° to the left or right to allow irrigation and suction of the entire circumference of the cavity. It is effective for a cavity of 9–10 cm in diameter, including the diameter of the burr-hole. Figure 2C is created by the author, Shoko M. Yamada. D: CT performed three days after surgery, showing definite brain expansion.

The endoscope used in the surgery was a rigid rod lens endoscope (Olympus, Hamburg, Germany) of diameter 4.0 mm with angles of 0º (A81000A), 30º (A81001A), and 70º (A81002A). A 0º endoscope was used for manipulation below the burr-hole and switched to 30º and 70º endoscopes as approached the limbus in the hematoma cavity (Figure 2Aa). After clots were removed as much as possible, the hard inner membrane was exposed; this was hard enough to be pushed by a sucker without damaging the brain cortex (Figure 2Ab), and no brain pulsation was recognized. After several incisions were made in the inner membrane by a sharp blade, expansion and pulsation of the brain were observed (Figure 2Ac, 2Ad). A straight suction device was used to remove the clots around the burr-hole, and an angled device was used to remove those at the edge of the cavity (Fujita Medical Instrument, Tokyo, Japan). The 4 cm end of the suction device has a 30º angle (Figure 2B); when the tip was inserted through the burr-hole into the hematoma cavity, it reached close to the limbus, allowing irrigation and suction and facilitating coagulation in case of bleeding (Figure 2C). Marked shrinkage of the hematoma cavity was confirmed by CT on the third postoperative day (Figure 2D). The patient recovered from hemiparesis (left MMT 5/5) and was discharged home independently on the 20th postoperative day. 

## Discussion

Although several treatments with good results have been reported for advanced organic CSDH, there is still no consensus on an established treatment. Some reports recommend craniotomy for organized CSDH instead of burr-hole irrigation and drainage [1,2]. Recently, for advanced organized CSDH, there have been multiple ongoing trials on middle meningeal artery embolization [6,7]. The general treatment for organized CSDH is burr-hole irrigation and drainage under local anesthesia, and some cases are resolved by this procedure. There may be differences between institutes in treatment strategies for cases of recurrent organized CSDH without brain expansion. For such patients, the authors have been exploring repeated irrigation and drainage using the previous burr-hole, and, in some cases, recurrent CSDH that appears to be incurable through simple drainage on MRI can be resolved completely with this repeated procedure. In cases where a third operation was required, we have performed a small craniotomy under general anesthesia, washed the hematoma cavity as much as possible, exposed the inner membrane, and made incisions in the membrane without membranectomy. It is considered that making incisions and tears in the inner membrane might be more hazardous [8], but our procedure can be performed more safely with the assistance of an endoscope. When incisions are made at the hard inner membrane, another layer of inner membrane or stiffened pia mater on the surface of the brain is identified in organized CSDH. By avoiding incisions in this deeper membrane, the cortex should never be at risk of damage. There have been some positive reports of craniotomy with membranectomy for advanced organized CSDH [1,2]. Cases of postoperative acute subdural hematoma, subarachnoid hemorrhage, intraventricular hemorrhage, pneumocephalus, and convulsive seizures have been reported, resulting in neurological deterioration [3-5]. The key steps of the endoscopic maneuver are to open a burr-hole at the center of the CSDH; remove clots and debris accumulating at the edge of the hematoma cavity that interfere with brain expansion (Figure 2Aa, 2Ab); make incisions randomly in the hard inner membrane, taking care not to damage the cerebral cortex; make incisions until brain expansion and pulsation are observed (Figure 2Ad); finally, apply a drain to the frontal side of the cavity. While it has been reported that longer periods of drainage are associated with reduced risk of CSDH recurrence [9,10], another report states that removing the drain in less than 24 hours does not change the recurrence rate [11]. In our experience, continued drainage is recommended for a few days, if necessary, until sufficient brain expansion is observed on CT. This is especially important for organized CSDH.

Several effective treatments for organic CSDH have been reported [1-8], endoscopic surgery being one of them. The area in which incisions can be made is narrower in burr-hole surgery than in craniotomy, and incisions into a strongly calcified inner membrane may be difficult to achieve through burr-hole. However, endoscopic burr-hole surgery is minimally invasive, can be performed under local anesthesia, and is effective, as demonstrated in our case. 

## Conclusions

There is no first-line treatment, per se, at the moment for advanced organized CSDH, although there are multiple treatment options. In fact, burr-hole irrigation is recommended nowadays only for the homogenous hypo- to iso-dense variant of CSDH by many neurosurgeons across the globe. But the authors recommend endoscopic surgery for CSDH cases that recur over short periods of time. Under the endoscope, the possibility of complete resolution can be increased by removing clots and making incisions in the hard inner membrane with a sharp blade until brain pulsation is observed. It is a viable treatment option with the need for large-scale pragmatic studies to prove safety and efficacy. 
